# Mapping the future of medicine through digital twins

**DOI:** 10.3389/fmmed.2026.1840371

**Published:** 2026-04-29

**Authors:** Frank Emmert-Streib, Shailesh Tripathi, Olli Yli-Harja

**Affiliations:** 1 College of Health and Life Sciences, Hamad Bin Khalifa University, Doha, Qatar; 2 Predictive Society and Data Analytics Lab, Faculty of Information Technology and Communication Sciences, Tampere University, Tampere, Finland; 3 Josef Ressel Centre for Data-Driven Business Model Innovation, University of Applied Sciences Upper Austria, Steyr, Austria; 4 Institute for Systems Biology, Seattle, WA, United States

**Keywords:** artificial intelligence, digital twins, dynamical systems, health data Science, health sciences, medical digital twins

## Abstract

Digital twins are rapidly gaining popularity in medicine and the health sciences, as they offer not merely a new method but a versatile paradigm capable of addressing a wide range of healthcare challenges. In this paper, we provide a focused survey of these developments and highlight promising approaches across several fields, including general health systems, cardiology, oncology, and mental health. We also discuss the key challenges and future directions for general medical digital twin systems.

## 1 Introduction

Digital twins are rapidly emerging as a transformative paradigm in medicine and the health sciences (Laubenbacher et al., 2024b; Armeni et al., 2022). Unlike conventional computational models, digital twins represent dynamical systems that can model both populations and individuals, integrating longitudinal data, physiological measurements, and clinical information to simulate real-world health processes (Emmert-Streib et al., 2025). This versatility enables them to address a wide range of challenges, from optimizing patient care and treatment strategies to modeling complex health systems (Katsoulakis et al., 2024; Emmert-Streib and Yli-Harja, 2022). In recent years, their applications have expanded across virtually all fields of medicine and the health sciences.

In this paper, we provide a focused survey of medical digital twins, highlighting notable examples, discussing their current limitations, and exploring promising directions for future research and clinical integration. We cover applications across multiple domains, including health systems, cardiovascular medicine, oncology, and mental health, to illustrate the breadth of current developments. Furthermore, we emphasize common methodological patterns and challenges, aiming to identify key factors that will shape the successful translation of digital twins into clinical practice. Finally, we outline opportunities for advancing the field through improved data integration, model validation, and interdisciplinary collaboration.

## 2 What are digital twins

Before we focus on applications of digital twins in medicine and the health sciences, we address briefly their definition. In simple terms, a digital twin is a continuously updated simulation model that learns over time (Wright and Davidson, 2020; Grieves, 2023). Often a digital twin is based on mechanistic models that can even provide causal information among system variables (Laubenbacher et al., 2024a; Emmert-Streib et al., 2024). A consequence thereof is that digital twins provide explainable models allowing not only to make predictions but leading to structural interpretations. For a more comprehensive discussion, the reader is referred to (Mazuz and Biswas, 2026; Singh et al., 2021) for foundational conceptual work.

## 3 Emergence of medical digital twins

To provide an overview of the literature on medical digital twins, we conduct a search using the Dimensions database of abstracts and citations (Adams et al., 2018). Compared to other databases, e.g., Scopus or WoS, Dimensions has two advantages. First, it provides a broader indexing of articles, similar to Google Scholar. Second, Dimensions gives a categorization of articles. Since for our investigation, publications in medicine and health sciences are of central importance, we use a categorization according to the Health Research Classification System (HRCS) developed by the UK Clinical Research Collaboration (UKCRC) Partners. In total, HRCS is subdivided into 21 separate categories which encompass all diseases, conditions and areas of health.

A search for the term “digital twin” in titles and abstracts within Dimensions yields 5,641 articles in total, including 2,908 identified from titles alone, distributed across 21 HRCS categories. Table 1 shows a summary of these articles. For clarity, this presents only the 17 categories with publications; no publications were found in blood, cancer and neoplasms, congenital disorders, ear. From Table 1 one sees that most articles fall within the broad category ’generic health relevance’ followed by cardiovascular and cancer and also the number of accumulated citations follows the same order. For the average number of citations (last column), a similar ranking is observed when restricting the analysis to categories with more than 50 publications. However, when including categories with fewer publications, ’renal and urogenital’ shows the highest citation impact across all fields. Notably, the ’inflammatory and immune system’ and respiratory categories also exhibit relatively high citation averages, indicating elevated interest in these areas.

**TABLE 1:** Publications and citations according to the Health Research Classification System (HRCS) about digital twins. The last column shows the mean number of citations per article as an indicator of interest in the published works.

HRCS	Publications	Citations	Citations (mean)
Generic health relevance	4,367	57,828	13.24
Cardiovascular	529	4,895	9.25
Cancer	322	2,898	9.00
Neurological	162	1,032	6.37
Mental health	121	884	7.31
Metabolic and endocrine	118	782	6.63
Infection	66	447	6.77
Musculoskeletal	65	280	4.31
Oral and gastrointestinal	47	401	8.53
Stroke	37	245	6.62
Inflammatory and immune system	36	366	10.17
Respiratory	30	301	10.03
Eye	18	29	1.61
Reproductive health and childbirth	18	52	2.89
Renal and urogenital	10	288	28.80
Skin	4	20	5.00
Injuries and accidents	1	19	19.00

**FIGURE 1:**
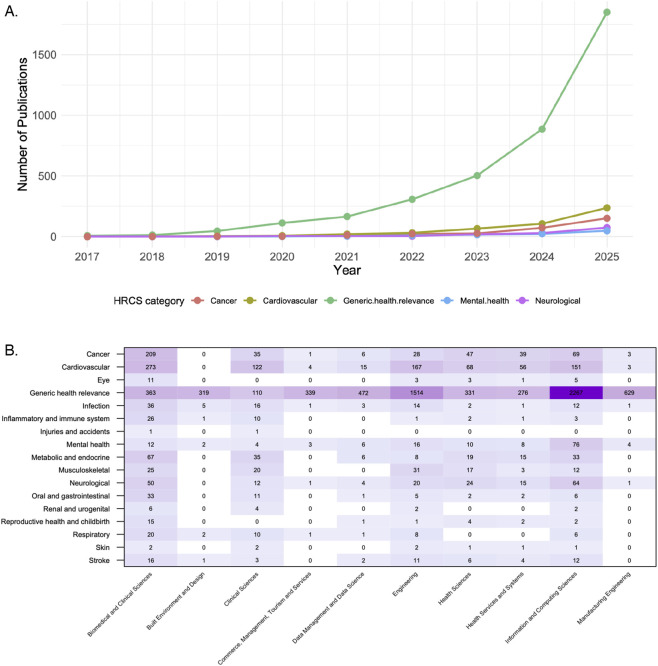
**(A)** Timeline of publications about digital twins in medicine and health sciences for the top 5 HRCS categories in Table 1. **(B)** Heatmap visualizing publication counts across different HRCS categories (y-axis) and fields of research (x-axis).

In Figure 1A, we present a timeline of published articles in the top five HRCS categories listed in Table 1 from 2017 to 2025. Although the concept of general digital twins began gaining attention in engineering and manufacturing around 2000 (Emmert-Streib et al., 2023), significant interest in medical digital twins has emerged only in recent years. In particular, the general relevance of digital twins in medicine and health sciences became apparent in 2019 within the generic health relevance category, while uptake in all other categories did not occur until 2023. This delay is understandable, given the challenges of translating the digital twin concept from engineering to medical applications.

To illustrate which research fields have contributed to this development, Figure 1B presents a heatmap of the distribution. The heatmap visualizes publication counts across different HRCS categories (y-axis) and fields of research (x-axis). Among all HRCS categories, Information and Computing Sciences dominates all other research fields followed by Engineering. Interestingly, while the Manufacturing Engineering field contributes a substantial number of publications across all HRCS categories, the majority are concentrated in ’generic health relevance’, suggesting a strong foundational focus with potential for further expansion into more specialized medical applications. Another interesting observation from Figure 1B comes from looking at Clinical Sciences. In this field, the most widely studied medical areas are cardiovascular, metabolic and endocrine, and cancer.

## 4 State-of-the-art medical digital twin applications

While medical digital twins are still in their infancy, several notable examples have already demonstrated their potential. In the following, we highlight studies across different fields that may provide guidance for future research.

### 4.1 General health systems

The study in (Penverne et al., 2024) applied a simulation-based digital twin of emergency medical communication centers (EMCCs) to evaluate organizational changes on accessibility. The digital twin models EMCC operations under decompartmentalized scenarios, showing that more flexible call distribution improves service quality. These results demonstrate how digital twins can provide an objective, data-driven tool to optimize emergency response systems and enhance performance.

A related study was conduced in (Kuruppu Appuhamilage et al., 2025) demonstrating the use of digital twins combined with discrete event simulation to optimize workflows in critical care units. A novel dual-layer architecture tracks both physical and conceptual entities in real time, with cloud-based monitoring of staff-performed tasks from observation forms. This approach highlights the potential of digital twins to enhance patient safety, operational efficiency, and precision care by aligning actual clinical practice with ideal workflows.

### 4.2 Human heart

Digital twin models of the human heart are among the most widely studied (Coorey et al., 2022; Sel et al., 2024; Fedele et al., 2023). In (Gerach et al., 2021), a multi-scale electro-mechanical whole heart model was presented that includes a coupled description of electrophysiology, mechanics, and a closed-loop model of circulation. This model allows the description of membrane kinetics, excitation-contraction coupling and active tension generation in the atria and the ventricles. Furthermore, the model can be personalized, e.g., by adjusting the characteristics of active tension or conduction velocity (CV).

A surrogate model for cardiac digital twins based on Latent Neural Ordinary Differential Equations (LNODEs) was introduced in (Salvador et al., 2024). The model learns pressure–volume dynamics from high-fidelity simulations while reducing computational complexity, enabling fast analyses such as sensitivity studies and parameter estimation with uncertainty quantification, and thereby significantly lowering the computational barrier for cardiac digital twins.

### 4.3 Oncology

The second most widely studied disease category for digital twin applications (see Table 1) is cancer (Hernandez-Boussard et al., 2021; Madhavan et al., 2021; Mollica et al., 2024). For example (Joslyn et al., 2024), developed a quantitative systems pharmacology (QSP)–based digital twin framework for TCR-engineered T cell therapy in patients with solid tumors, including metastatic HPV-associated epithelial cancers and pancreatic cancer. These digital twins capture the kinetics of infused and endogenous T cells across blood, lymph nodes, tumor, and peripheral tissues. Patient-matched digital twins reproduce observed T cell dynamics and show that stem cell–like memory T cells (Tscm) are key determinants of expansion and persistence, explaining inter-patient variability. In silico simulations suggest that Tscm enrichment enhances persistence and enables lower dosing, providing a quantitative tool to optimize T cell therapies and guide future clinical strategies.

Another digital twin application in oncology was presented in (Zhang et al., 2025), which introduced a model to predict treatment-induced toxicity in patients with acute myeloid leukemia (AML). This approach employs a mechanistic model of venetoclax and azacitidine therapy, informed by measurements of neutrophil counts and blast percentages from blood samples. This study demonstrated that continuously updating the digital twin with new data enhances its predictive performance over time.

### 4.4 Mental health

Digital twins have also shown significant promise in advancing research and applications in mental health (VE, 2025; Spitzer et al., 2023; Maes, 2022). For example (Abilkaiyrkyzy et al., 2024), proposed a digital twin–based framework implemented as a chatbot system that analyzes the mental status of individuals and provides personalized feedback based on the severity of their condition. The system leverages pre-trained BERT models fine-tuned on the E-DAIC dataset to detect varying severity levels, achieving high accuracy and usability. By creating virtual representations of mental states, this approach addresses barriers such as stigma, limited accessibility, and cost, enabling scalable screening, early intervention, and actionable insights for mental health professionals.

A similar concept is presented in (Yücel et al., 2026), which introduces a digital twin–inspired mental health monitoring system (MTWIN) that created a virtual representation of a patient’s psychological state. MTWIN integrates personal and medical information, wearable sensor data, environmental factors, and emotion predictions from a machine learning model to track and visualize mental health progression. The system provides patients with actionable feedback on daily habits and gives healthcare professionals a historical overview of treatment response.

Finally, the Digital Alzheimer’s Disease Diagnosis (DADD) digital twin model was introduced in (Amato et al., 2025) for early Alzheimer’s disease diagnosis. DADD uses personalized biomarkers derived from non-invasive EEG recordings to reconstruct patient-specific neurodegeneration, capturing synaptic and connectivity decline. The model predicts cerebrospinal fluid biomarker positivity and clinical conversions with high accuracy, outperforming standard EEG methods. By enabling robust, non-invasive assessment of preclinical AD, DADD demonstrates the potential of digital twins to transform early diagnosis and prognostic evaluation in Alzheimer’s disease.

## 5 Challenges and future directions

Despite the promising capabilities of medical digital twins, several challenges need to be addressed to make them applicable for widespread clinical and medical use.

- Data availability and quality: Limited access to high-quality, longitudinal, and multimodal patient data is a major obstacle, as digital twins are largely data-driven. In particular, repeated measurements over time are required to establish high-quality digital twins that can be personalized to individual patients. In this context, data from wearables may provide valuable complementary information alongside clinical, genomic, and other data sources.
- Model validation, benchmarks, and clinical trust: Ensuring the accuracy and robustness of digital twins remains a major barrier to clinical adoption and requires rigorous, task-specific validation against conventional approaches. Establishing standardized benchmarks is an open challenge, including data quality, model validation, clinical utility, interoperability, and uncertainty quantification Corral-Acero et al. (2020); Katsoulakis et al. (2024); David et al. (2024); Sel et al. (2025); Pash et al. (2025). In particular, Sel et al. (2025) highlights the need for real-world benchmark datasets that capture the dynamic nature of clinical settings, as well as standardized evaluation methods to assess analytical accuracy and support reliable clinical decision-making.
- Computational complexity: High-resolution multimodal data, increasing model complexity, and the use of physics-informed neural networks (PINNs) and high-fidelity models (e.g., organ-level or tumor growth simulations) are computationally intensive and require large-scale computing resources for digital twin modeling Venkatesh et al. (2022); Olawade et al. (2026). This computational cost makes real-time model updating challenging. To address this, approaches such as hybrid physics-machine learning surrogate models and edge-optimized architectures can be employed to reduce latency and improve model accuracy. Furthermore, defining “real-time” in terms of disease-specific time scales can help reduce data requirements and computational demands, as many physiological changes occur over much longer periods rather than at high-frequency resolutions.
- Regulatory issues and data privacy: Regulatory issues represent a major challenge for the deployment of medical digital twins, as existing frameworks are not yet fully adapted to dynamic, data-driven models. Key concerns include ensuring safety, reliability, and clinical validity, particularly when models are continuously updated with new patient data. In addition, questions around data privacy, ownership, and informed consent must be addressed, especially when integrating sensitive health information from multiple sources.

Finally, we discuss several promising yet demanding directions for future research on medical digital twins, highlighting key opportunities to advance their development and clinical integration.

- Slow progressing diseases: Given that digital twins rely on time series data collected over extended periods, slowly progressing diseases may be especially well suited for this approach. For example, Alzheimer’s disease and Parkinson’s disease involve gradual changes over many years (Patel et al., 2024), making them ideal for longitudinal modeling and long-term data integration. Sensor data from wearables can provide rich time series measurements to capture subtle, disease-induced changes, offering valuable insights into disease progression. Unlike conventional approaches, digital twins can operate with cost-efficient data, are continuously updated, and are designed for long-term use.
- Integrating electronic health records: While patients may initially present with a single disease, many develop multiple conditions over the years. This information is captured in electronic health records (EHRs). Rather than creating separate disease-specific digital twins, integrating these data allows the construction of a comprehensive digital twin representing the underlying patient, capturing the interactions and progression of multiple coexisting conditions.
- Health economics and sustainable health: It is important to recognize that the concept of a digital twin is not limited to individual patients but can also be applied to the broader health system. Public health systems may be represented by digital twins that model expenditures across diseases, resource allocation, and patient care pathways. Such system-level digital twins may simulate the impact of policy changes, new interventions, or demographic shifts on healthcare costs and outcomes. In this way, they may help identify inefficiencies, optimize resource distribution, and forecast future demands. This supports more informed decision-making and contributes to both the economic sustainability of healthcare systems and the long-term improvement of population health.
- Interoperable digital twin with connectivity to other DTs for complex medical tasks: In medicine, many complex problems, such as multi-organ diseases, integrated patient care, or hospital workflow optimization, may not be captured by a single digital twin alone. Similar to industrial digital twins, which may leverage interoperability and connectivity for efficiency and scalability Li et al. (2025); Human et al. (2023); David et al. (2024), medical digital twins may also benefit from connected frameworks. By enabling multiple specialized medical DTs, such as heart, liver, metabolism, and mental health, to share data, models, and simulations, clinicians and researchers may address complex, multi-factorial conditions and coordinated care pathways more effectively. Future development may therefore require system-level integration frameworks that support cross-discipline DT interactions, co-simulation, and collaborative learning across different medical DTs.

## 6 Conclusion

Medical digital twins are an emerging paradigm with the potential to profoundly transform medicine due to their ability to adapt to a wide range of problems. In this paper, we reviewed their early development and highlighted proofs of concept across several fields, including general health systems, cardiology, oncology, and mental health. We also discussed key challenges and outlined future directions for medical digital twins.
